# ER stress and calcium-dependent arrhythmias

**DOI:** 10.3389/fphys.2022.1041940

**Published:** 2022-11-08

**Authors:** Shanna Hamilton, Dmitry Terentyev

**Affiliations:** ^1^ Department of Physiology and Cell Biology, The Ohio State University, Columbus, OH, United States; ^2^ Dorothy M. Davis Heart and Lung Research Institute, College of Medicine, The Ohio State University Wexner Medical Center, Columbus, OH, United States

**Keywords:** cardiac arrhythmia, sarcoplasmic reticulum, redox, er stress, calcium signaling, ryanodine receptor, sarco/endoplasmic reticulum Ca-ATPase, unfolded protein response

## Abstract

The sarcoplasmic reticulum (SR) plays the key role in cardiac function as the major source of Ca^2+^ that activates cardiomyocyte contractile machinery. Disturbances in finely-tuned SR Ca^2+^ release by SR Ca^2+^ channel ryanodine receptor (RyR2) and SR Ca^2+^ reuptake by SR Ca^2+^-ATPase (SERCa2a) not only impair contraction, but also contribute to cardiac arrhythmia trigger and reentry. Besides being the main Ca^2+^ storage organelle, SR in cardiomyocytes performs all the functions of endoplasmic reticulum (ER) in other cell types including protein synthesis, folding and degradation. In recent years ER stress has become recognized as an important contributing factor in many cardiac pathologies, including deadly ventricular arrhythmias. This brief review will therefore focus on ER stress mechanisms in the heart and how these changes can lead to pro-arrhythmic defects in SR Ca^2+^ handling machinery.

## Introduction

The sarcoplasmic reticulum (SR) is a membrane-bound structure within cardiomyocytes, analogous to the endoplasmic reticulum (ER), with a major function as a Ca^2+^ storage organelle. This function is critical for excitation-contraction coupling, whereby release of large amounts of SR Ca^2+^ *via* ryanodine receptors (RyR2s) drives cardiac contraction (Bers, 2002). Reuptake of this Ca^2+^ *via* the SR Ca^2+^ ATPase (SERCA2a) leads to cardiac relaxation. The magnitude and frequency of SR Ca^2+^ release is tightly regulated by sarcolemmal electrical activity. In turn, Ca^2+^ released from the SR affects sarcolemmal membrane potential by activating electrogenic Na^+^/Ca^2+^ exchanger and modulating function of several ion channels and transporters, i.e., L-type Ca channels or small conductance Ca^2+^-activated K^+^ channels etc. (Hamilton et al., 2021) Compromised SR Ca^2+^ homeostasis due to altered RyR2 and SERCA2a function has been linked to arrhythmogenesis in many inherited and acquired cardiovascular diseases (Bers, 2002; Hamilton and Terentyev, 2018; Hamilton et al., 2021). Enhanced RyR2 activity and spontaneous Ca^2+^ release promotes pro-arrhythmic disturbances in sarcolemma membrane potential called early and delayed after-depolarizations (EADs and DADs, respectively), contributing to the initiation of triggered activity (Landstrom et al., 2017). Additionally, diminished SR Ca^2+^ release during systole due to depleted stores often ascribed to reduced SERCA2a activity and/or enhanced RyR2 function reduces cardiac contractility (Zima et al., 2014). Furthermore, abnormal intracellular Ca^2+^ handling in the form of beat-to-beat variations of Ca^2+^ release magnitude can contribute to arrhythmia substrate *via* cardiac alternans promoting reentry (Edwards and Blatter, 2014). Understanding molecular mechanisms governing regulation of SR Ca^2+^ homeostasis is therefore paramount in order to develop therapies to prevent arrhythmogenesis and sudden cardiac death.

In addition to its role in intracellular Ca^2+^ handling, the SR/ER is the site of quality control machinery that orchestrates synthesis, folding and degradation of new proteins (Glembotski, 2014). The oxidative environment of the organelle is critical for protein homeostasis, and is tightly controlled by protein disulfide isomerases (PDI) and oxidoreductase enzymes (Otsu et al., 2006; Zito, 2013; Ramming et al., 2015; Zito, 2015). The PDIs enable transfer of electrons by oxidoreductases such as ERO1α to oxygen, facilitating the formation of disulfide bridges and folding of proteins into functional, three-dimensional structures (Ramming and Appenzeller-Herzog, 2012). This also results in the production of ROS that can modify SR/ER proteins, including RyR2 and SERCA2a, altering Ca^2+^ release and reuptake (Chin et al., 2011).

As H_2_O_2_ is a byproduct of oxidative protein folding, the SR is increasingly recognized as a significant source of ROS within the cardiomyocyte, in addition to well-established sources such as NADPH oxidase 2 or mitochondria (Bertero and Maack, 2018; Hamilton et al., 2022). While stress of the SR/ER invokes initially adaptive pathways to restore protein-folding capacity and upregulate antioxidant machinery, chronic ER stress invokes maladaptive pathways of the UPR, including excessive ROS production and degradation of protein mRNA. Growing evidence suggests ER stress is implicated in multiple cardiovascular disease pathologies associated with Ca^2+^-dependent arrhythmias, including hypertrophy, heart failure, ischemia/reperfusion and diabetic cardiomyopathy (Glembotski, 2008; Dalal et al., 2012; Kassan et al., 2012; Xu et al., 2012; Liu et al., 2014; Wiersma et al., 2017; Freundt et al., 2018; Liu H. et al., 2021; Liu M. et al., 2021; Sirish et al., 2022).

Here, we briefly review how chronic SR/ER stress contributes to aberrant remodeling of intracellular SR Ca^2+^ handling and the development of ventricular Ca^2+^-dependent cardiac arrhythmias.

## The SR as the major source of Ca^2+^ for cardiomyocyte contraction

As a highly specialized form of the ER, the SR is a membranous, intricate network of tubules and cisternae designed to regulate Ca^2+^. Despite only assuming 3.5% of cellular volume, the SR serves as the major Ca^2+^ storage organelle and provides up to 60%–90% of Ca^2+^ that activates contractile machinery in the cytosol (Bers, 2002). The enormous buffering capacity of the SR is afforded by the high capacity, low-affinity Ca^2+^-binding protein Calsequestrin (CASQ2) (Gyorke et al., 2009).

During systole, large amounts of Ca^2+^ are released from the SR *via* ryanodine receptors (RyR2), substantially increasing cytosolic Ca^2+^ from 100 to 500 nM (Williams et al., 2011). Although RyR2 is the primary SR Ca^2+^ release channel, inositol 1,4,5-trisphosphate receptors (IP_3_R2) are also Ca^2+^ release channels of the SR, but their functional role in healthy ventricular cardiomyocytes remains somewhat controversial (Domeier et al., 2008; Kockskamper et al., 2008). During diastole, the majority of this Ca^2+^ is sequestered back to the SR by SERCA2a in an ATP-dependent process (Bers, 2002). Pumping activity of SERCA2a is negatively regulated by interaction with inhibitory protein phospholamban (PLB). Inhibition mediated by PLB is relieved by the post translational modification phosphorylation, enhancing the affinity of SERCA2a for Ca^2+^ and increasing SR Ca^2+^ uptake.

Dynamic and reversible posttranslational modifications of both RyR2 and SERCa2a are critical for the grading of SR Ca^2+^ release to respond to changes in metabolic demands (Bers, 2002; Zima and Blatter, 2006). Many laboratories, including our own, have established RyR2 activity is augmented by reversible redox posttranslational modifications, increasing the sensitivity of the channel to intra-SR (luminal) Ca^2+^ (Terentyev et al., 2008; Belevych et al., 2012; Bovo et al., 2012; Cooper et al., 2013; Hamilton et al., 2020). Disulfide bridge formation, glutathionylation, nitrosylation and phosphorylation by reactive oxygen species (ROS)-sensitive Serine/Threonine kinase CaMKII have all been demonstrated to increase channel activity (Boraso and Williams, 1994; Eager and Dulhunty, 1998; Salama et al., 2000; Hidalgo et al., 2002; Mattiazzi et al., 2015; Kim et al., 2017; Bovo et al., 2018; Hegyi et al., 2021). Additionally, SERCA2a has several cysteine residues susceptible to oxidative modification (Sharov et al., 2006), and direct oxidation at cytosolic Cysteine 674 was demonstrated to decrease Ca^2+^ pump activity in cardiomyocytes (Lancel et al., 2009; Hobai et al., 2013). Redox-dependent phospholamban oligomerization also relieves inhibition of SERCA2a and this contributes to enhanced activity (Froehlich et al., 2008; Sivakumaran et al., 2013).

It is well established that redox modifications on the cytosolic side of the SR can modify Ca^2+^ channel activity. Thus it is not surprising that the vast majority of RyR2 Cysteines identified to have redox-sensitive potential reside in the huge, cytosolic structure of the channel (Pessah and Feng, 2000; Aracena-Parks et al., 2006; Nikolaienko et al., 2018). Modification of these residues, some of which reside within binding sites of accessory proteins Calmodulin (CaM) and FKBP, may also interfere with protein-protein interactions on the cytosolic side of the channel (Balshaw et al., 2001; Fauconnier et al., 2010; Shan et al., 2010; Mattiazzi et al., 2015). For example, redox-mediated cross-linking of RyR2 at the cytosolic N-terminus has been recently linked to increased dissociation of CaM from the channel and enhanced SR Ca^2+^ leak (Nikolaienko et al., 2020). Less redox-sensitive Cysteines are present in the smaller luminal region of RyR2, and the relevance of oxidative modification at these sites, as well as the effect of oxidative stress on luminal protein-protein interactions, remains poorly defined. Information regarding the functional relevance of SERCA2a luminal Cysteines is also limited, although two conserved residues are present in the longest luminal loop that protrudes well into the oxidizing SR environment (Marino et al., 2015).

## The SR as a major site of cardiomyocyte protein homeostasis

In the heart, hypertrophy of cardiomyocytes underlies adaptation to both physiological and pathological stresses (Wilkins and Molkentin, 2004), and as the site of protein synthesis and folding, the SR plays a major role in determining whether hypertrophy is adaptive or maladaptive. Proper protein synthesis, folding and degradation is critical to hypertrophic growth, and dysregulation can lead to the accumulation of toxic, misfolded proteins detrimental to cellular function (Glembotski, 2008; Hetz et al., 2015; Oakes and Papa, 2015). Therefore, protein homeostasis requires a fine-tuned and elaborate protein quality control system, with inherent plasticity to respond to growth stimuli, and pro-survival mechanisms to prevent cell death.

As part of this quality control system, the ER stress response is triggered when ER homeostasis—Ca^2+^ levels, redox status or protein-folding capacity–is disturbed (Hetz et al., 2015). This results in an initial accumulation of misfolded proteins within the ER, as well as the induction of chaperone proteins including those of the heat shock family such as glucose-regulated protein 78/binding immunoglobulin protein (GRP78/BiP), and lectin-like chaperones, such as calreticulin and calnexin. As excessive accumulation of proteins is detrimental to the cell, chaperone induction serves as an adaptive mechanism to help refold proteins, or target them for degradation.

Chaperone GRP78 is an important activator of the unfolded protein response (UPR), complex signaling cascades that relieve ER stress by increasing protein-folding capacity and decreasing protein folding load (Bertolotti et al., 2000; Glembotski, 2008). The UPR is initiated by activation of three ER-transmembrane sensors: activating transcription factor 6α/beta (ATF6) (Yoshida et al., 1998), inositol requiring enzyme 1 α/beta (IRE1) (Cox et al., 1993), and PKR-like ER kinase (PERK) (Shi et al., 1998). In conditions of efficient protein folding, luminal domains of ATF6, IRE1, and PERK are locked in an inactive state by interaction with ER-resident chaperone GRP78. When efficient protein folding is disrupted and misfolded proteins begin to accumulate, GRP78 dissociates and initiates each UPR signaling cascade (Bertolotti et al., 2000; Shen et al., 2005; Glembotski, 2008).

The ATF6 signaling cascade has mainly adaptive functions, decreasing the amount of proteins within the ER/SR and increasing protein-folding capability (Glembotski, 2014). GRP78-mediated activation and translocation of ATF6 to the Golgi leads to cleavage of the cytosolic domain by proteases SP1 and SP2. The cleaved, active fragment then translocates to the nucleus, activating b-Zip transcription factors that upregulate the expression of protein folding chaperone, as well as genes involved in ER-associated degradation of terminally misfolded proteins (ERAD) (Meusser et al., 2005).

Unlike ATF6, IRE1 does not re-locate to the Golgi, but instead acts within the cytosol. Upon ER stress, dimerization and autophosphorylation of IRE1 drives a conformational change that activates an endoribonuclease domain within the protein (Sidrauski and Walter, 1997; Calfon et al., 2002). This domain excises an intron from b-Zip transcription factor X-box binding protein 1 (XBP-1), leading to an XBP1 transcript with a new, and active, open reading frame. XBP-1 then moves to the nucleus and binds elements required for rotein folding and ERAD. In a mechanism known as regulated IRE1-dependent decay (RIDD), IRE1 activity leads to degradation of microRNAs, ribosomal RNA and ER-localized mRNA (Bhattarai et al., 2021). Additionally, ubiquitination of IRE1 leads to recruitment of TNF receptor-associated factor 2 (TRAF2) and activation of the pro-apoptotic apoptosis signal-regulating kinase/c-jun N-terminal kinase (ASK/JNK) cascade (Hasnain et al., 2012). Together, this helps to rebalance protein synthesis and protein folding, enabling cell survival.

Similar to IRE1, luminal dissociation of GRP78 upon ER stress leads to activation of PERK by homodimerization and autophosphorylation. Activated PERK phosphorylates eukaryotic initiation factor 2α (eIF2α), transiently reducing its efficiency as a translation initiator. This inhibits ribosomes, degrades mRNA and stops protein synthesis, preventing overload within the SR and aiding in resolving ER stress (Bertolotti et al., 2000). Conversely, although PERK activation mostly reduces translation efficiency, phosphorylation of eIF2α leads to increased levels of activator of transcription factor 4 (ATF4). Upregulation of ATF4 increases gene expression of ER chaperones and directs an antioxidant response, facilitating greater protein folding capacity (Glembotski, 2008).

Although ER stress and activation of UPR is initially a pro-survival mechanism, chronic ER stress drives a switch to a maladaptive and terminal UPR that favors cellular apoptosis (Han et al., 2013). For example, during prolonged ER stress, ATF4 activates CCAAT/enhancer-binding protein homologous protein (CHOP), which in turn inhibits the expression of anti-apoptotic BCL-2 and leads to translocation of Bax from the cytosol to the mitochondria, promoting cell death (Gotoh et al., 2004). Moreover, transcriptional activation of CHOP also induces cell death by promoting oxidation in the stressed ER, activating the ER oxidase ERO1α (Marciniak et al., 2004) and depleting intracellular glutathione (McCullough et al., 2001). Although it has important physiological roles within the ER in disulfide bond formation, ERO1α is also a significant producer of ROS in the form of H_2_O_2_, and excessive upregulation promotes a hyper-oxidizing SR (Ramming et al., 2015; Zito, 2015). This in turn can drive redox modifications of SR proteins such as ion channels involved in calcium homeostasis, linking SR redox state with SR Ca^2+^ handling.

## SR/ER stress and cardiac arrhythmias

It is well established that UPR is activated in various cardiac pathologies associated with increased arrhythmic risk. For example, in failing human and animal hearts, increased levels of AT4, CHOP and GRP78 are reported (Hamilton et al., 2022). We have recently demonstrated upregulation of CHOP and downstream oxidoreductase ERO1α in hypertrophic rat myocytes (Hamilton et al., 2022). Diabetic cardiomyopathy is also associated with ER stress and activated UPR (Liu et al., 2014; Liu et al., 2021), as is cancer chemotherapy-induced cardiotoxicity (Kerkela et al., 2006).

**FIGURE 1:**
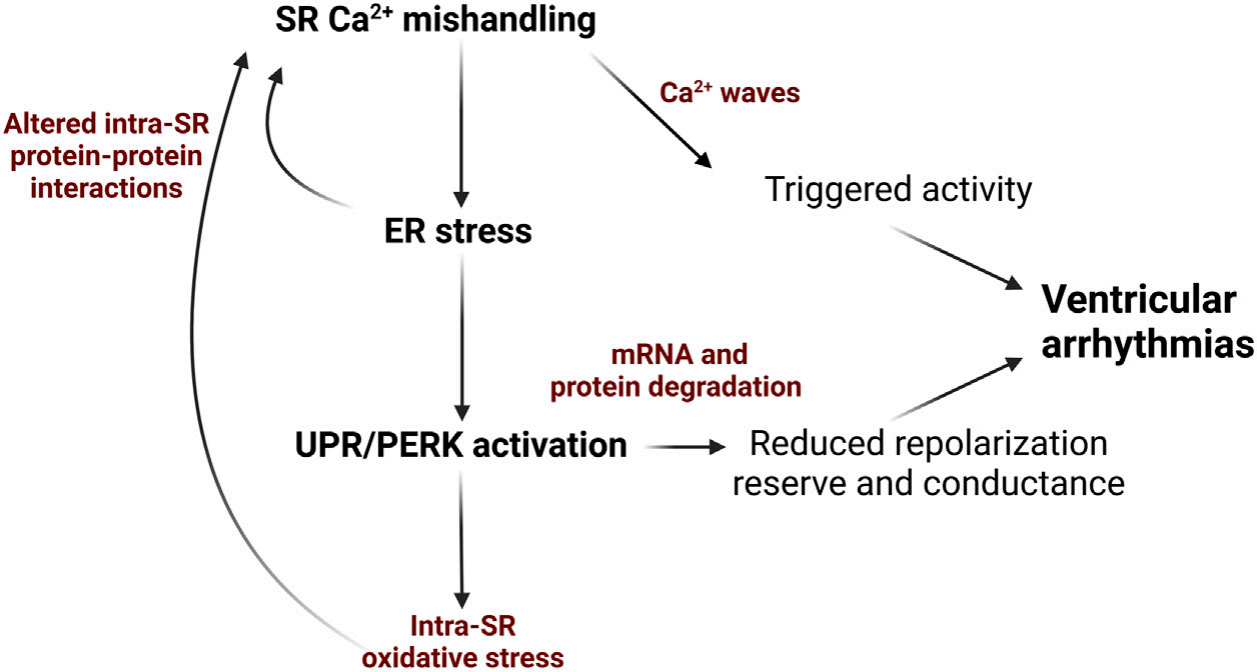
Schematic depicting how ER stress and the UPR can exacerbate SR Ca^2+^ mishandling *via* in cardiovascular disease and contribute to Ca^2+^-dependent ventricular arrhythmogenesis. Diminished SR Ca^2+^ content due to decreased SERCA2a activity and/or increased RyR2 activity evokes ER stress and UPR that promotes ROS production mRNA and protein degradation and impair protein-protein interactions in Ca^2+^-handling complexes. This results in decreased repolarization reserve and slowing conduction due to reduction in K^+^ and Na^+^ ion channels in the plasmalemma. Additionally, further enhancement of RyR2 activity and reduction in SERCA2a activity promotes generation of pro-arrhythmic spontaneous SR Ca^2+^ waves. Image created with Biorender.com.

Given the physical and functional link between SR protein and Ca^2+^ homeostasis, there is an emerging view that chronic SR/ER stress is linked to increased arrhythmic risk *via* perturbed redox status within the SR, as well as downregulation of cardiac ion channel proteins (Figure 1).

### Dysregulation of redox homeostasis

Increasing evidence suggests redox changes on the luminal side of the SR, in addition to the cytosolic side, can modulate SR Ca^2+^ homeostasis in cardiomyocytes. Work of the Ron laboratory revealed that mice with loss-of-function of luminal oxidoreductase ERO1α were protected from pressure-overload induced heart failure (Chin et al., 2011). As a downstream effector of the PERK UPR branch, this enzyme is known to be upregulated during ER stress (Zito, 2013). Importantly, ERO1α-deficient cardiomyocytes had reduced Ca^2+^ transient amplitude in comparison to controls (Chin et al., 2011), implicating ER stress and altered SR Ca^2+^ release in this protection.

We recently demonstrated a novel regulatory axis for RyR2-ROS modulation involving ERO1α and luminal binding of RyR2 by protein ERp44 (Hamilton et al., 2022). The PDI-like protein ERp44 was previously reported to inhibit Ca^2+^ release channel inositol triphosphate receptor type 1 (IP_3_R1) by direct protein-protein interaction, and this association was redox-dependent (Anelli et al., 2002; Higo et al., 2005; Wang et al., 2014). Of note, ERp44 interacts with an IP3R1 region that has sequence homology to the RyR2 second intraluminal loop, and using a multi-pronged approach, we revealed ERp44 directly interacts with RyR2 in this region *via* disulfide bond formation (Hamilton et al., 2022). This protein-protein interaction is redox-dependent and exerts a stabilizing influence on channel function. Under conditions of increased ER stress such as cardiac hypertrophy, upregulation of ERO1α removes ERp44 from the RyR2 channel complex, contributing to spontaneous Ca^2+^ release from the SR and increased risk for Ca^2+^-dependent arrhythmias. Importantly, inhibition of ERO1α was able to restore intra-SR redox potential, attenuate aberrant RyR2 activity and thus prevent ventricular arrhythmogenesis at the whole heart level. We also demonstrated that application of reducing agent dithiothreitol could not reverse the ER stress-associated dissociation of ERp44 from RyR2. Reducing agents typically improve intracellular Ca^2+^ handling, but offer only partial recovery (Terentyev et al., 2008). This may be explained by their mechanism of action, preventing stabilizing disulfide bridge formation between SR Ca^2+^ handling proteins such as RyR2 and ER stress components such as ERp44. Our study therefore highlighted the importance of understanding luminal protein-protein interactions involved in SR Ca^2+^ release, to better inform therapeutic approaches when treating Ca^2+^-dependent arrhythmias.

Protein homeostasis needs to be tightly regulated, and requires several systems to prevent excessive ROS accumulation from oxidative protein folding by enzymes such as ERO1α. The SR/ER localized Peroxiredoxin (PRDX4) reduces cellular redox stress by using H_2_O_2_ generated by ERO1α to generate disulfide bridges (Zito, 2013). This serves to protect the cell from accumulation of misfolded proteins. A recent report posits downregulation of SR-specific Peroxiredoxin-4 (PRDX4) increases oxidative stress in cardiac fibroblasts and contributes to heart failure (Ibarrola et al., 2018). Additionally, PRDX4 knockdown accelerated pressure-overload induced cardiac hypertrophy in mice (Zhang et al., 2021), linking a disease-associated imbalance of H_2_O_2_ –producing and–detoxifying systems with cardiac dysfunction.

Although the functional relevance of cytosolic Cysteine 674 oxidation in SERCA2a is well established (Lancel et al., 2009), less is known about redox regulation of luminal residues of SERCA2a in cardiomyocytes. All SERCA isoforms contain a conserved pair of Cysteines in the fourth luminal loop, which form a disulfide bridge (Daiho et al., 2001). The Zito laboratory have previously demonstrated that selenoprotein N (SEPN1), an SR/ER resident protein with redox function, enhances SERCA2 activity (2a and 2b) in skeletal muscle by reducing the two luminal Cysteines that are hyperoxidized by ERO1α-generated H_2_O_2_ (Marino et al., 2015). Although not yet tested in native cardiomyocytes, authors also confirmed that SEPN1 covalently associates with SERCA2a expressed in HEK293 cells through the equivalent residues, Cysteine-875 and Cysteine-C887 (Marino et al., 2015). Later studies revealed that the EF-hand domain in SEPN1 is sensitive to changes in luminal Ca^2+^ concentration, with low levels driving a conformational change which activates the protein as a reductase. This consequently regulates the redox status of SERCA2, thus serving as a feedback mechanism to replenish luminal Ca^2+^ stores (Chernorudskiy et al., 2020).

Work of the Kranias laboratory revealed a link between oxidative stress and degradation of SERCA2a in ischemia-reperfusion injury (Lam et al., 2013; Bidwell et al., 2018). In diseased cardiomyocytes, they demonstrated decreased HS-1-associated protein X-1 (HAX-1), a protein involved in apoptosis. Through interaction with protein chaperone Hsp90, HAX-1 inhibits IRE1 signaling at the SR/ER. Ablation of HAX-1 in adult mice hearts resulted in SERCA2a degradation, while HAX-1 overexpression restored SERCA2a levels and improved contractility. Authors demonstrated underlying mechanisms for this included increases in SERCA2a oxidation and reactive oxygen production at the ER/SR, through direct interaction of HAX-1 with NOX4.

Aberrant lipid synthesis also appears to disrupt SR/ER homeostasis, and upregulation of UPR has been associated with metabolic syndrome (Smith et al., 2022). Heart failure with preserved ejection fraction (HFpEF) is a multifactorial disease, initiated by a variety of factors including hypertension and obesity, and characterized by preserved systolic function, reduced ability to relax and importantly, oxidative stress (Roh et al., 2022; Smith et al., 2022). Defective Ca^2+^ handling, including altered activity of RyR2 and SERCA2a, have been reported as contributing to diastolic dysfunction in multiple models of HFpEF (Reil et al., 2013; Kilfoil et al., 2020; Miranda-Silva et al., 2020). Gaining traction in recent HFpEF research is a “two-hit” mouse model fed high fat diet in combination with nitric oxide synthase inhibitor, which recapitulates some of the concomitant metabolic and hypertensive phenotype of the human disease (Schiattarella et al., 2019). Interestingly, Schiattarella et al. (2019) found that in hearts from both mice and humans with HFpEF, expression of the spliced and active form of XBP1 was reduced, attributable to increased S-nitrosylation of IRE1. Authors also demonstrated that downregulation of XBP1 in this model drives downstream lipid accumulation, and cardiomyocyte-specific overexpression was able to ameliorate the HFpEF phenotype (Schiattarella et al., 2019). It appears that downregulation of the IRE1/XBP pathway of UPR is unique to the pathogenesis of HFpEF, in comparison to other cardiovascular diseases. Additionally, reduced levels of SR/ER glutathione peroxidase 4 were reported in this two-hit mouse model (Kitakata et al., 2021). These findings reveal crosstalk of signaling pathways between UPR and metabolism as well as perturbed SR redox in HFpEF, with potential implications for the function of SR Ca^2+^ handling proteins and arrhythmogenesis.

In addition to oxidative modifications, RyR2 and SERCA2a are susceptible to reversible phosphorylation, serving as a central mechanism to grade Ca^2+^ release from the SR (Terentyev and Hamilton, 2016). The serine-threonine protein phosphatase calcineurin (PP2B), a focal regulator of cardiac hypertrophy in cardiovascular disease (Wilkins and Molkentin, 2004), is also established as a modulator of RyR2 and SERCA2a function (Bandyopadhyay et al., 2000; Münch et al., 2002). The UPR was linked to activity of calcineurin by the finding that calcineurin inhibitor RCAN1 was inducible by the ATF6 gene (Liu et al., 2014). By reducing calcineurin-mediated dephosphorylation of NFAT, ATF6-mediated activation of RCAN1 contributes to reducing protein load and serves as a mechanism to regulate the extent of cardiac hypertrophy. However, increased inhibition of calcineurin by RCAN1 or other inhibitors in cardiac pathologies can be detrimental to SR Ca^2+^ homeostasis, given that maximum phosphorylation or incomplete dephosphorylation of RyR2 can result in increased channel activity (Hiemstra et al., 2014; Liu et al., 2014; Terentyev and Hamilton, 2016).

### Dysregulation of protein homeostasis

The UPR is reported to regulate the expression levels of many ion channels involved in excitation-contraction coupling including SR Ca^2+^ handling proteins (Liu et al., 2022), linking ER stress to increased arrhythmic risk. Knockdown of calnexin, an ER quality control chaperone, led to increased ER stress and apoptosis induced by caspase-3 and -9, in parallel with increased expression of LTCC and reduced expression of SERCA2a (Bousette et al., 2014). Reduced expression of SERCA2a was also observed in mice with cardiac ablation of HAX-1, where this maneuver elicited increased luminal ROS production and oxidation of SERCA2a, driving its proteolysis and degradation (Lam et al., 2013; Bidwell et al., 2018).

A body of work from the Dudley laboratory has demonstrated that under ER stress, the PERK branch of the UPR contributes to the downregulation of other cardiac ion channels in a variety of settings, including human heart failure and in human induced pluripotent stem cell–derived cardiomyocytes (hiPSC-CMs) treated with UPR activator tunicamycin (Liu and Dudley, 2014; Liu et al., 2021). The group showed reductions in Nav1.5, Kv4.3, and Kv1.5 in human heart failure were PERK-dependent, suggestive that ion channel downregulation is induced by ER stress and contributes to cardiac arrhythmogenesis (Liu et al., 2021). The UPR was shown to be activated in mice with myocardial infarct, in parallel with downregulation at the mRNA and protein level of Nav1.5, Cav1.2, Kv4.3, Kir2.3 and Kv1.5 ion channels, and increased arrhythmic risk. Importantly, inhibition of PERK in mice prevented downregulation of these channels, attenuated aberrant electrical remodeling, reduced ventricular arrhythmia inducibility and improved survival after myocardial infarct. Therefore, it is evident that the UPR can contribute to proarrhythmic cardiac remodeling. This is suggestive that targeting ER stress components in cardiovascular disease can serve as an anti-arrhythmic strategy, improving both SR redox and protein folding homeostasis and improving intracellular Ca^2+^ handling.

## Discussion

The SR/ER protein homeostasis system is a convergence point for signaling pathways. As the SR/ER processes a vast array of proteins, dysregulated UPR and thus redox and protein homeostasis may affect proteins involved in Ca^2+^-dependent arrhythmogenesis, and uncovering these molecular mechanisms is critical for designing new therapeutic approaches.

Targeting ER stress in the heart has shown promise in treating cardiovascular disease (Liu and Dudley, 2014; 2018). Chemical chaperones such as TUDCA and 4-PBA suppress ER stress and offer cardiac protection, attenuating intracellular Ca^2+^ mishandling in hypertrophic rat myocytes and alleviating obesity-induced myocardial contractile dysfunction (Ceylan-Isik et al., 2011; Hamilton et al., 2022). PERK inhibition with GSK260614, atorvastatin and apelin-13 was shown to prevent ventricular arrhythmia and reduce apoptosis in MI and ischemia/reperfusion mouse models (Song et al., 2011; Tao et al., 2011).

Despite promising results, targeting ER stress as a druggable node in cardiovascular disease is complex, given the fine balance between mild activation of UPR fostering protection vs. chronic activation driving cellular damage. Targeting the correct, harmful UPR branch in the specific disease setting is critical while other arms are activated to suppress detrimental SR protein loading (Toko et al., 2010; Liu et al., 2022; Wang et al., 2022). For example, activation of the ATF6α branch of UPR is considered an adaptive responder to SR/ER stress and offers protective effects in many cardiac disease settings (Glembotski et al., 2020). *Ex vivo* hearts from transgenic mice with ATF6α activation were protected from damage induced by ischemia reperfusion (Martindale et al., 2006). At the cellular level, overexpression of ATF6α attenuated ROS generation and protected against apoptosis induced by ischemia/reperfusion (Jin et al., 2017). Small molecule therapies to activate ATF6α in the context of cardiac disease are beginning to be explored in mice with evidence of improved contractile function after ischemia reperfusion injury (Blackwood et al., 2019), but this has yet to be extended to other larger animal models or other cardiac pathologies. Furthermore, IRE1α overexpression preserved cardiac function and reduced the inflammatory response to pressure overload in mice (Steiger et al., 2018), suggestive that transient ER stress signaling of the IRE1α branch can confer protective effects to the heart.

Conversely, inhibition of the PERK UPR branch has demonstrated cardioprotective effects in multiple animal models of cardiac disease, including myocardial infarct and ischemia reperfusion injury (Tao et al., 2011; Gao et al., 2013; Liu et al., 2021). Additionally, inhibiting downstream effectors of the PERK branch has also proved to be cardioprotective (Chin et al., 2011; Liu et al., 2013; Hamilton et al., 2022). It has been shown that drug-mediated inhibition of CHOP can attenuate ventricular remodeling after myocardial infarct in rats by reducing apoptosis (Liu et al., 2013). We have recently demonstrated the antiarrhythmic effects of inhibiting ERO1α, an oxidoreductase downstream of PERK and CHOP in a model of cardiac hypertrophy (Hamilton et al., 2022). However, when targeting any ER stress/UPR branch component, global inhibition *in vivo* is likely to have detrimental side effects, given the physiological role of ER stress in cell types and organs across the body. Genetic inhibition of PERK and downstream effectors may offer advantages over pharmacological approaches, as off-target effects in organs other than the heart can be avoided. The effects of chronic cardiac-specific inhibition of UPR components needs to be thoroughly explored and studied in larger animal models of cardiovascular disease.

The complexity of ER stress as a therapeutic approach is also underscored by our limited understanding of redox-dependent protein-protein interactions within the SR. While reducing agents and antioxidants reduce proarrhythmic spontaneous SR Ca^2+^ release and partially restore SR Ca^2+^ content in isolated cardiomyocytes (Mochizuki et al., 2007; Terentyev et al., 2008; Belevych et al., 2012; Kim et al., 2017), this has never translated to the clinic, with no improvement in propensity for arrhythmias or other outcomes for heart failure patients (Sawyer, 2011; van der Pol, 2019; Szyller et al., 2022). Reduction of reactive cysteines on both the cytosolic and luminal side of RyR2 does not completely stabilize the channel complex, and does not allow for interactions with proteins such as ERp44 that depend on disulfide bridge formation (Hamilton et al., 2022). It is also likely SERCA2a interacts with luminal proteins of the ER stress system, as is observed with other pump isoforms in different cell types (Li and Camacho, 2004; Marino et al., 2015).

There is a paucity of information regarding the effects of the UPR on the Ca^2+^ buffering capacity of the SR/ER. A direct interaction between Calsequestrin, a major SR Ca^2+^ buffer, and IRE1α pooled at the junctional SR has been demonstrated in cardiomyocytes (Wang et al., 2019). There is also evidence that in heart failure, a condition accompanied by impaired ability of SR to retain Ca^2+^ (Zima et al., 2014) and ER stress (Hamilton et al., 2022), Calsequestrin processing and trafficking can be impaired (Jacob et al., 2013). However, the effects of this interaction on Ca^2+^ buffering and thus activity of RyR2 and cardiac arrhythmias are yet to be elucidated. On the other hand, it is well established that impaired ability to retain Ca^2+^ in the SR is a key contributor to ER stress highlighted in SERCA2a KO mice (Liu et al., 2011), pressure-overload induced hypertrophy (Nie et al., 2019), and ischemia-reperfusion studies (Valverde et al., 2010; Cai et al., 2012). Importantly, reduction in SR Ca^2+^ leakage not only improves cardiac function in ischemia/reperfusion challenged hearts (Bovo et al., 2018; Mariangelo et al., 2022), but is capable to reduce ER stress as well (Mariangelo et al., 2022). Of note, ER stress inhibition in aged or ischemia-reperfusion challenged hearts improves mitochondrial function (Chen et al., 2017; Chen et al. 2020; Chen et al. 2021). The works from multiple groups including our own established a direct connection between the pro-arrhythmic increase in RyR2 activity and accelerated ROS production by mitochondria (Belevych et al., 2011; Cooper et al., 2013; Joseph et al., 2016; Bovo et al., 2018; Hamilton et al., 2018; Hamilton et al., 2020; Hamilton et al., 2021; Liu H et al., 2021), thereby linking ER stress with an increase in arrhythmic potential.

It does appear that directly targeting ER stress *via* genetic approaches, as opposed to untargeted cell-wide pharmacological approaches, is a more promising approach to attenuate Ca^2+^-dependent ventricular arrhythmias. Reducing CHOP activity was shown to attenuate effects of pressure overload in TAC mouse models (Fu et al., 2010). Inhibition of PERK decreases ventricular arrhythmias and improves cardiac function in MI mouse models (Liu et al., 2018; Liu et al., 2021). Of note, PERK is upstream of ERO1α in the UPR. We have demonstrated that both pharmacological and genetic approaches to reduce ERO1α activity, which is upregulated in cardiovascular disease, reduces spontaneous SR Ca^2+^ release and arrhythmic risk without perturbing the fine-tuned redox balance of the SR (Hamilton et al., 2022).

**FIGURE 2:**
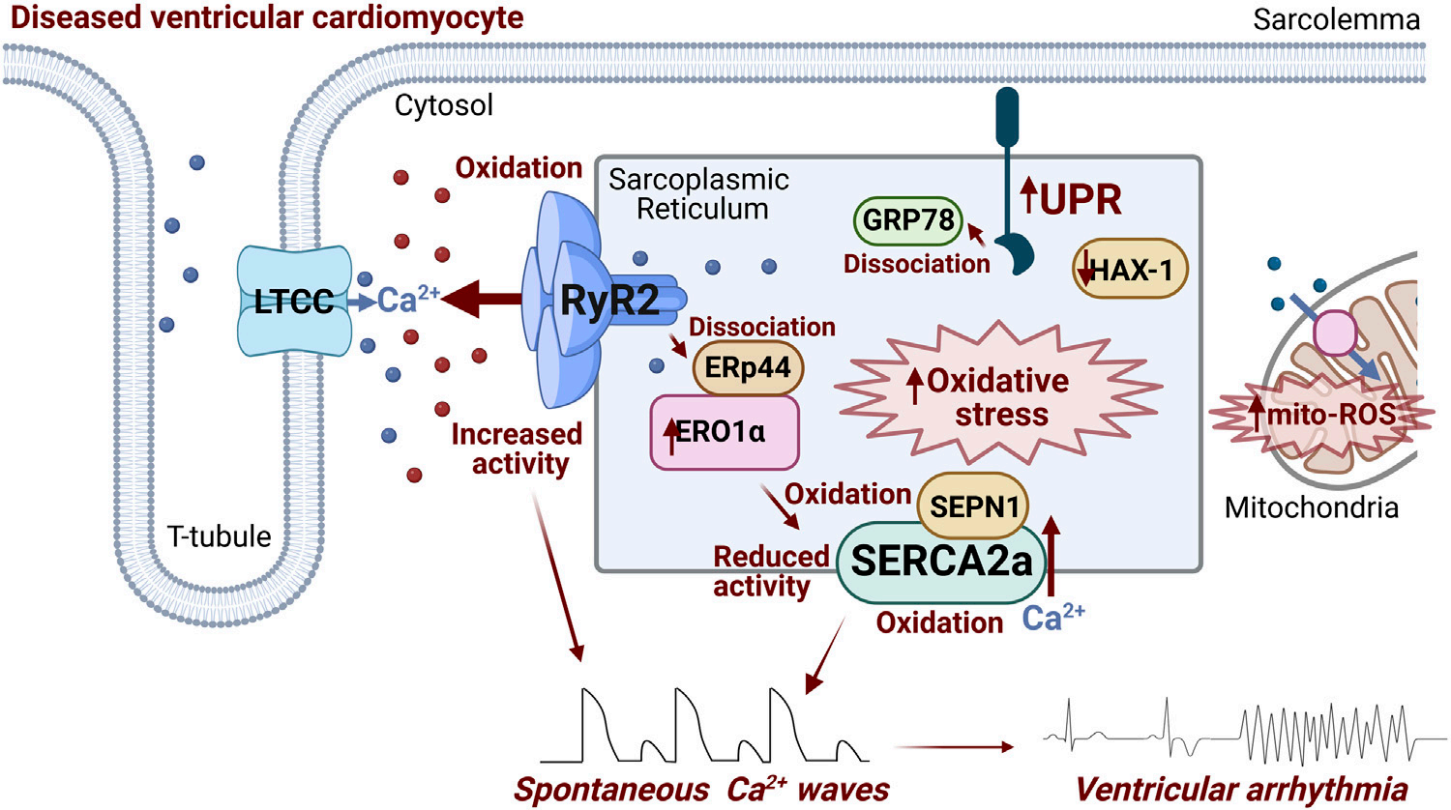
Schematic summarizing ER stress-associated modulation of RyR2 and SERCA2a occurring in diseased ventricular cardiomyocytes that contributes to arrhythmogenesis. Activation of ER stress and the UPR increases ERO1α expression/activity, promoting generation of ROS. ERO1α dissociates ERp44 from the RyR2 complex, increasing channel activity. Additionally, intra-SR oxidative stress promotes RyR2 oxidation at cytosolic face of the channel, exacerbating its hyperactivity. Putative oxidation of two intraluminal SERCA2a cysteines and Cysteine 674 at cytosolic face decreases the activity of transporter. SEPN1 serves as a redox sensor of SERCA2a, with low levels of luminal Ca^2+^ activating the protein as a reductase, and mitigating increased oxidation levels. Downregulation of HAX-1 in diseased hearts leads to less inhibition of the UPR by the protein, driving downstream SERCA2a oxidation and protein degradation. Image created with Biorender.com.

To conclude, ER stress and the UPR can exacerbate SR Ca^2+^ mishandling *via* RyR2 and SERCA2a (Figure 2) in cardiovascular disease and contribute to Ca^2+^-dependent arrhythmogenesis, through dysregulated SR oxidative status, perturbed luminal protein-protein interactions and protein homeostasis (Figure 1). Given the burden of Ca^2+^-dependent cardiac arrhythmias and sudden cardiac death, uncovering novel axes of intraluminal interactions between SR/ER stress proteins and SR Ca^2+^ handling proteins, whether direct or indirect through redox modification, may reveal new therapeutic approaches for Ca^2+^-dependent arrhythmias.
